# Prognostic Significance of Prolonged Corrected QT Interval in Acute Ischemic Stroke

**DOI:** 10.3389/fneur.2021.759822

**Published:** 2021-12-20

**Authors:** Sung-Ho Ahn, Ji-Sung Lee, Young-Hak Kim, Mi-Sook Yun, Jung-Hee Han, Soo-Young Kim, Min-Gyu Park, Kyung-Pil Park, Dong-Wha Kang, Jong S. Kim, Sun U. Kwon

**Affiliations:** ^1^Department of Neurology, Research Institute for Convergence of Biomedical Science and Technology, Pusan National University School of Medicine, Pusan National University Yangsan Hospital, Busan, South Korea; ^2^Division of Cardiology, Asan Medical Center, College of Medicine, University of Ulsan, Seoul, South Korea; ^3^Clinical Research Center, Asan Medical Center, College of Medicine, University of Ulsan, Seoul, South Korea; ^4^Division of Biostatistics, Research Institute for Convergence of Biomedical Science and Technology, Pusan National University School of Medicine, Pusan National University Yangsan Hospital, Busan, South Korea; ^5^Department of Neurology, Asan Medical Center, College of Medicine, University of Ulsan, Seoul, South Korea

**Keywords:** QTc interval, ischemic stroke, mortality, electrocardiography, comorbidities

## Abstract

**Background and Purpose:** The aim of this study was to determine the relationship between the heart rate-corrected QT (QTc) interval and the risk of incident long-term mortality in patients with acute ischemic stroke (AIS), considering the impact of sex differences on clinical characteristics, outcomes, and QTc intervals.

**Methods:** We analyzed prospectively registered data included patients with AIS who visited the emergency room within 24 h of stroke onset and underwent routine cardiac testing, such as measurements of cardiac enzymes and 12-lead ECG. QTc interval was corrected for heart rate using Fridericia's formula and was stratified by sex-specific quartiles. Cox proportional hazards models were used to examine the association between baseline QTc interval and incident all-cause death.

**Results:** A total of 1,668 patients with 1,018 (61.0%) men and mean age 66.0 ± 12.4 years were deemed eligible. Based on the categorized quartiles of the QTc interval, cardiovascular risk profile, and stroke severity increased with prolonged QTc interval, and the risk of long-term mortality increased over a median follow-up of 33 months. Cox proportional hazard model analysis showed that the highest quartile of QTc interval (≥479 msec in men and ≥498 msec in women; hazard ratio [HR]: 1.49, 95% confidence interval [CI]: 1.07–2.08) was associated with all-cause death. Furthermore, dichotomized QTc interval prolongation, defined by the highest septile of the QTc interval (≥501 ms in men and ≥517 m in women: HR: 1.33, 95% CI: 1.00–1.80) was significantly associated with all-cause mortality after adjusting for all clinically relevant variables, such as stroke severity.

**Conclusions:** Prolonged QTc interval was associated with increased risk of long-term mortality, in parallel with the increasing trend of prevalence of cardiovascular risk profiles and stroke severity, across sex differences in AIS patients.

## Introduction

The QT interval on surface ECG represents ventricular repolarization time, and prolongation of the heart rate-corrected QT (QTc) interval is associated with functional re-entry, torsade de pointes, and sudden death (1). Aside from its direct association with the risk of fatal arrhythmia, prolonged QTc interval is associated with increased risks of mortality and incident cardio-cerebrovascular disease in both high-risk individuals (2, 3) and the general population (4–6).

Although prolonged QTc interval is prevalent and one of the most common ECG abnormalities in patients with acute ischemic stroke (AIS) (7, 8), the clinical utility of QTc interval duration in AIS remains limited due to complex mechanism. Apart from neurally mediated autonomic dysregulation leading to prolong the QTc interval (9, 10), other factors prevalent in patients with stroke may contribute to QTc interval prolongation (11); these include atherosclerotic risk factors, cardiac diseases, electrolyte imbalance, and certain drugs. Furthermore, sex differences should be considered as one of the most decisive factors determining abnormal QTc interval prolongation (12), but also, as a potential confounder leading to a disproportionate distribution of QTc prolonging factors, such as age, atherosclerotic risk factors, cardiovascular and cerebrovascular risk profiles, and even medications (13).

The present study was designed to determine the relationship between QTc interval and the risk of incident long-term mortality in patients with AIS while considering the impact of sex differences on clinical characteristics, outcomes, and QTc intervals.

## Materials and Methods

### Study Population

We analyzed prospectively registered data included patients with AIS who visited the emergency room within 24 h of symptom onset and were admitted to the Asan Medical Center between May 2007 and December 2011. While in the emergency room, all patients underwent routine cardiac testing, such as measurements of cardiac enzymes and 12-lead ECG investigations were performed on admission according to the stroke protocols in our center, which abide by the 2007 guidelines (14). Patients underwent additional cardiac evaluations by a cardiologist if they were suspected to suffer from acute coronary syndrome during the evaluation at the emergency department. Then, patients were excluded if (1) they were diagnosed with the concomitant acute coronary syndrome (15) at admission, or (2) their brain images or ECGs were of poor quality, or (3) they had a complete bundle branch block (QRS interval > 120 ms), ventricular rhythm, or pacemaker-paced rhythm. The study protocol was approved by the Institutional Review Board of the Asan Medical Center, which waived the requirement for informed consent because of the registered data analysis design of this study.

### Patient and Public Involvement in the Study

Patients and the public were not involved and were not applicable in this study.

### Electrocardiogram Analysis and QT Interval Duration

All patients underwent a 12-lead ECG (GE Healthcare, Waukesha, WI) at admission, with the results processed using the Marquette 12SL ECG Analysis Program. The resultant 12-lead ECG waveforms were uploaded in digital form and interpreted by a cardiologist according to a modified version of the Minnesota code (16). The QT interval was defined as the time duration between the earliest QRS onset to the latest T-wave offset in the 12 ECG leads. For calculation of QTc interval, Fridericia's formula was used because it has been regarded as being appropriate for calculating QTc interval in patients with tachycardia or bradycardia (17), or AF due to the beat-to-beat variability in the RR interval (18, 19). QTc intervals were calculated by a specialized cardiologist and stratified by quartiles for each sex.

### Data Acquisition

Clinical data were obtained from the patients' electronic medical records. These included demographic characteristics, conventional risk factors for stroke, comorbidities such as a previous history of stroke and cardiac comorbidities. The latter included ischemic heart disease (IHD), defined as a medical history of IHD or evidence of prior IHD on admission by 12-lead ECG; atrial fibrillation (AF), defined as a history of AF, evidence of AF on admission by 12-lead ECG, and newly diagnosed AF after admission; ventricular hypertrophy (VH), defined as a medical history of hypertrophic cardiomyopathy or evidence of VH on admission by 12-lead ECG; and congestive heart failure (CHF), defined as a history of cardinal manifestations and treatment for heart failure. Other factors recorded included chronic kidney disease (CKD), defined as an estimated glomerular filtration rate <60 ml/min/1.73 m^2^ on admission; and active cancer, defined as cancer within 6 months prior to enrollment, any treatment for cancer within the previous 6 months, or recurrent or metastatic cancer (20). Neurological status was determined using the National Institutes of Health Stroke Scale (NIHSS) (21), which assessed stroke severity and the specific locations of insular cortical lesions.

### Collection of Mortality Data

Follow-up information for patients was obtained using the national death certificate data from the Korean National Statistical Office until 31 December 2012. The nationwide official data for death certificates produced by the Korean National Statistical Office are updated annually. Deaths were classified according to the *International Classification of Diseases, Tenth Revision* (22). Causes of death were classified as stroke (ICD codes: I60–I69), cardiac causes (ICD codes: I20–I25 or I30–I52), malignancies (ICD codes: C00-C96), and other causes.

### Statistical Analysis

Baseline characteristics were compared according to the distribution of QTc intervals, which were categorized by quartiles for each sex. Continuous variables were expressed as means ± SDs or medians (interquartile ranges [IQR]) and compared by ANOVA tests. Categorical variables were expressed as numbers (%) and compared by Chi-square tests.

Multivariate Cox proportional hazards models were used to determining the relationship between the quartiles of sex-specific QTc intervals and all-cause death in all patients and each sex. Hazard ratios (HRs) are reported with 95% CIs. Model 1 included adjustments for age, sex, conventional risk factors for stroke, comorbidities, and all laboratory results, whereas Model 2 included all the variables in Model 1, as well as NIHSS scores for estimating the HR of quartiles and dichotomized QTc interval prolongation. Variables were included in a stepwise method based on our previous studies, with a consideration of the strong impact of neurologic deficits on long-term mortality as well as QTc-interval prolongation (23, 24). The timing of events according to QTc interval was assessed by the Kaplan–Meier method, with curves compared by log-rank tests. In addition, the prognostic value of dichotomized QTc interval prolongation, defined by cut-offs according to the highest median, tertile, quartile, quintile, sextile, septile, octile, and decile of sex-specific QTc intervals, was compared with the generally adopted cut-offs for community-based QTc interval prolongation (i.e., ≥450 ms in men and ≥460 ms in women) (25) to determine the optimal prognostic cut-off value for QTc interval prolongation. The discriminatory power of the models was estimated using Harrell's C-statistics. All reported *p*-values were two-sided, with *p* < 0.05 considered statistically significant. All statistical analyses were performed using SPSS for Windows version 17.0 (SPSS Inc., Chicago, IL).

## Results

### Baseline Characteristics

**Figure 1:**
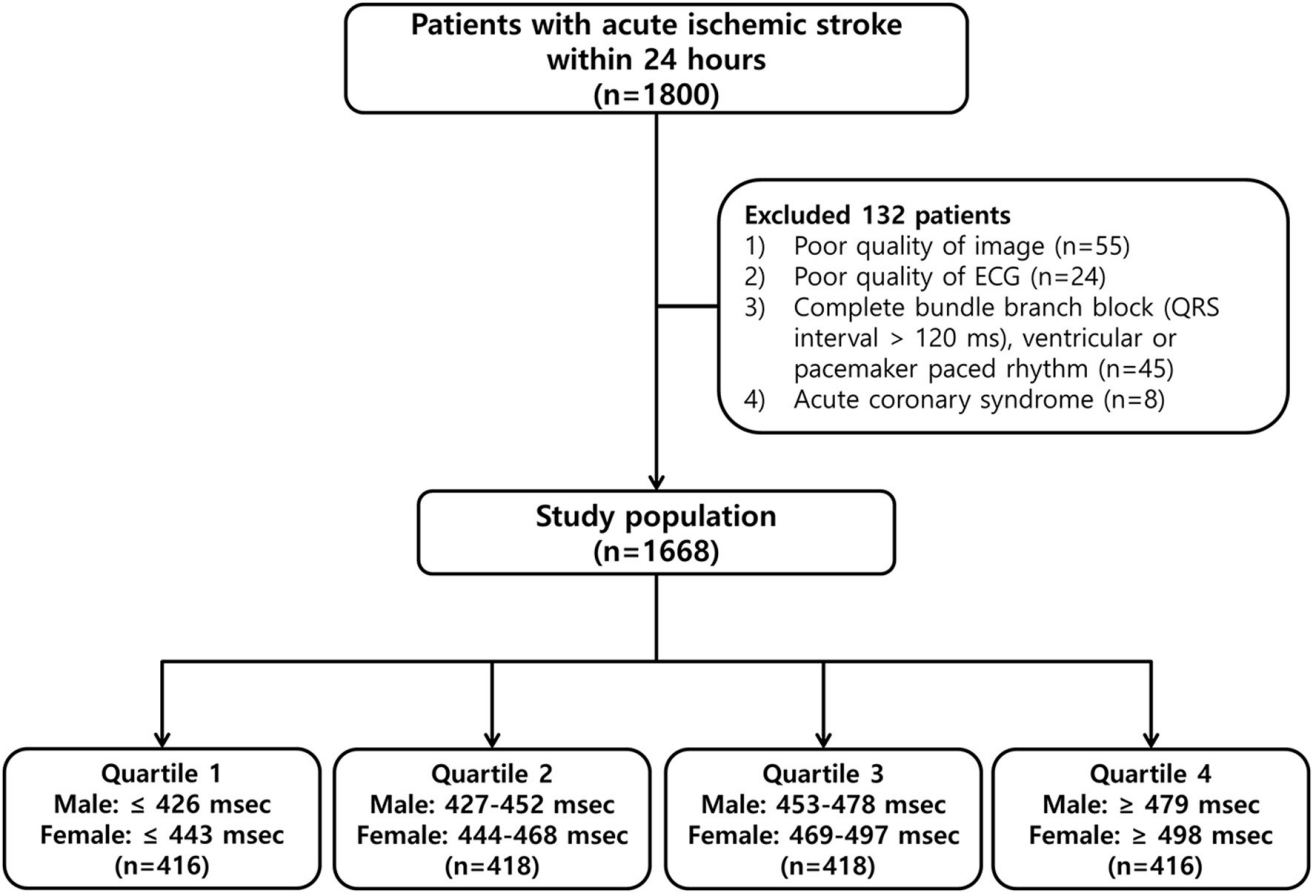
Flowchart of the patient selection process and classification by quartiles of the corrected QT (QTc) interval.

We included 1,668 patients in this study (Figure 1), 1,018 (61.0%) men and 650 (39.0%) women, of mean age 66.0 ± 12.4 years (range, 24–96 years). Their mean QTc interval was 462.3 ± 43.9 ms (range, 343–809 ms), and the sex-specific mean QTc intervals were 455.2 ± 41.8 ms (range, 311–654 ms) in men and 473.3 ± 44.7 ms (range, 357–809 ms) in women (Supplementary Figure 1).

Table 1 summarizes the baseline characteristics in patients stratified by the quartiles of sex-specific QTc intervals. Patients in higher quartiles of QTc interval were tended to be older, and were more likely to have rapid heart rates and a lower proportion of people with a sinus rhythm on baseline ECG, hypertension, and comorbidities, such as AF, CHF, and CKD; and had higher white blood cell (WBC) counts and glucose and possibly C-reactive protein (CRP) concentrations than patients with lower quartiles of QTc interval. In addition, patients in higher quartiles of QTc interval had higher NIHSS scores than patients with lower quartiles of QTc interval.

**Table 1:** Characteristics according to the corrected QT (QTc) intervals.

	Quartiles of QTc interval^*^				
**Variable**	**Q1 (*n* = 416)**	**Q2 (*n* = 418)**	**Q3 (*n* = 418)**	**Q4 (*n* = 416)**	***P-*value^†^**
Age (years)	64.9 ± 12.2	65.8 ± 12.4	66.8 ± 12.4	66.7 ± 12.5	0.10
Male	254 (61.1)	255 (61.0)	255 (61.0)	254 (61.1)	1.00
**Baseline ECG information**					
Heart rate	66.3 ± 13.2	73.9 ± 13.5	79.2 ± 14.8	91.2 ± 19.7	<0.01
Normal sinus rhythm	300 (72.1)	312 (74.6)	268 (64.1)	227 (54.6)	<0.01
**Risk factors**					
Hypertension	243 (58.4)	257 (61.5)	281 (67.2)	280 (67.3)	0.02
Diabetes mellitus	92 (22.1)	102 (24.4)	106 (25.4)	117 (28.1)	0.25
Hyperlipidemia	90 (21.6)	95 (22.7)	101 (24.2)	92 (22.1)	0.84
Current smoking	125 (30.0)	132 (31.6)	134 (32.1)	123 (29.6)	0.84
**Comorbidities**					
Prior stroke	96 (23.1)	107 (25.6)	118 (28.2)	112 (26.9)	0.37
IHD	51 (12.3)	61 (14.6)	58 (13.9)	59 (14.2)	0.78
AF	106 (25.5)	86 (20.6)	122 (29.2)	158 (38.0)	<0.01
VH	107 (25.7)	98 (23.4)	99 (23.7)	125 (30.0)	0.11
CHF	29 (7.0)	29 (6.9)	48 (11.5)	67 (16.1)	<0.01
CKD	33 (7.9)	56 (13.4)	61 (14.6)	73 (17.5)	<0.01
Comorbid cancer	19 (4.6)	27 (6.5)	23 (5.5)	19 (4.6)	0.56
**Laboratory results**					
WBC (10^3^/uL)	7.8 ± 2.6	7.9 ± 2.8	8.3 ± 2.6	8.9 ± 3.3	<0.01
PLT (10^3^/uL)	220.8 ± 60.6	222.8 ± 68.6	218.8 ± 64.0	226.8 ± 75.6	0.39
Hb (g/dL)	13.9 ± 1.8	13.8 ± 1.9	13.9 ± 1.9	13.9 ± 2.2	0.96
Glucose (mg/dL)	136.4 ± 53.4	144.6 ± 55.6	151.0 ± 65.0	149.4 ± 50.9	<0.01
Albumin (g/dL)	3.8 ± 0.4	3.8 ± 0.4	3.8 ± 0.4	3.8 ± 0.5	0.67
HDL (mg/dL)	42.7 ± 12.1	43.0 ± 11.6	43.1 ± 12.0	43.3 ± 11.9	0.89
LDL (mg/dL)	111.2 ± 36.8	108.0 ± 34.0	109.2 ± 32.7	105.1 ± 34.3	0.10
Homocysteine (mmol/mL)	14.5 ± 7.4	14.9 ± 7.5	15.4 ± 7.3	14.3 ± 5.8	0.14
CRP (mg/dL)	0.5 ± 1.6	0.7 ± 2.1	0.7 ± 2.0	0.9 ± 2.7	0.07
**Characteristics of stroke**					
NIHSS score	3 [1–7]	4 [2–8]	5 [2–10]	5 [3–12]	<0.01

* *QTc cut-off points between quartiles 1 and 2, 2 and 3, and 3 and 4 were 427, 453, and 479 ms, respectively, for men and 444, 469, and 498 ms, respectively, for women*.

*Variables are presented as mean ± SD, median [interquartile range], or number (%)*.

*AF, atrial fibrillation; CHF, congestive heart failure; CKD, chronic kidney disease; CRP, C-reactive protein; Hb, hemoglobin; HDL, high-density lipoprotein; IHD, ischemic heart disease; LDL, low density lipoprotein; NIHSS, National Institutes of Health Stroke Scale; PLT, platelet; VH, ventricular hypertrophy; WBC, white blood cell*.

† *p-values were calculated by Pearson chi-square test or ANOVA test, as appropriate*.

**Figure 2:**
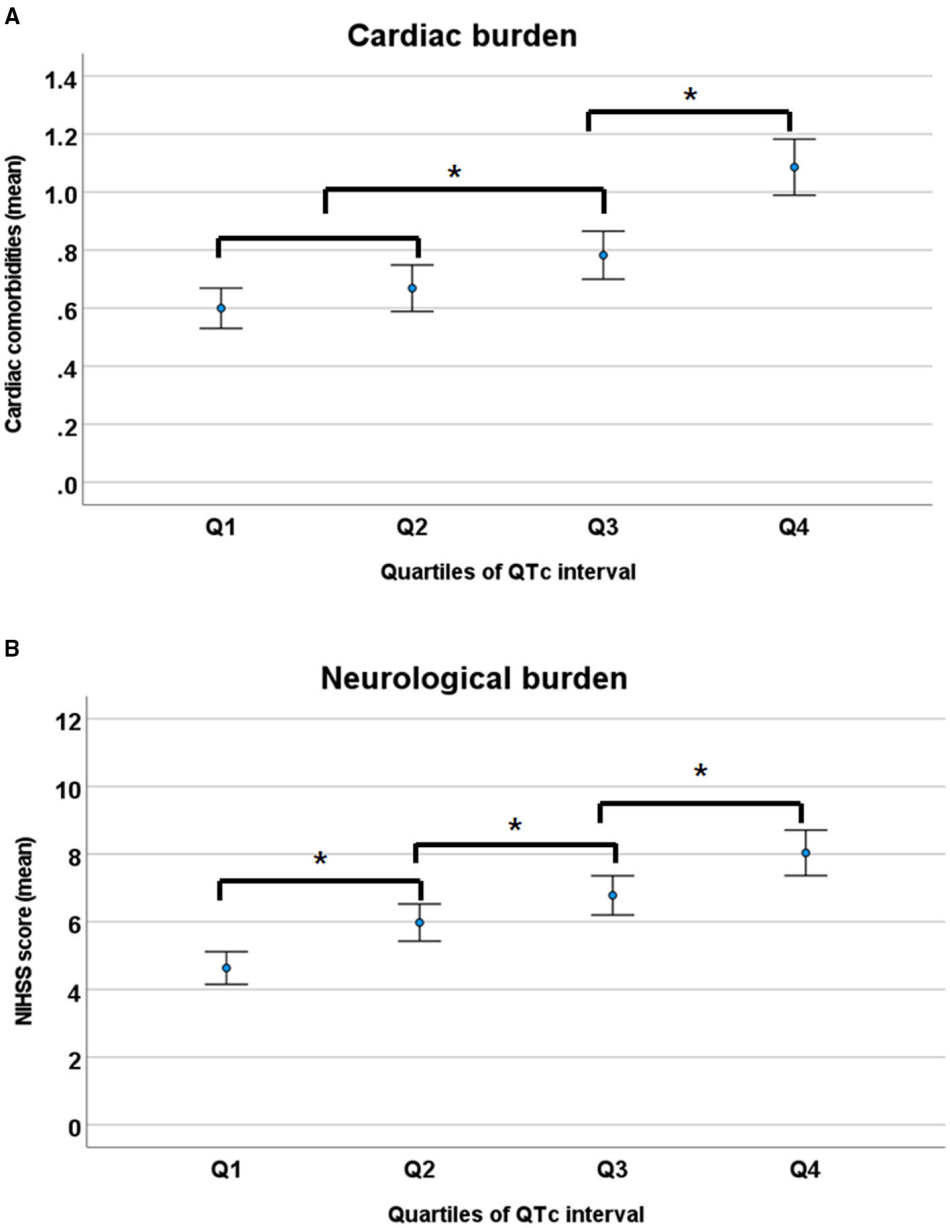
Cardiac **(A)** and neurological burden **(B)** according to the quartiles of QTc intervals. NIHSS, National Institutes of Health Stroke Scale. *p < 0.05 by ANOVA with Duncan *post-hoc* test.

To assess the relationship of QTc interval to the overall burden of cardiac and neurologic conditions, the cardiac burden was defined as the mean number of cardiac comorbidities, such as IHD, AF, VH, and CHF; and the neurological burden was defined as mean NIHSS score, representing stroke severity. Overall, cardiac and neurologic burdens gradually increased with prolonged QTc intervals (Figure 2).

### QTc Interval for Prediction of Long-Term Mortality

**Figure 3:**
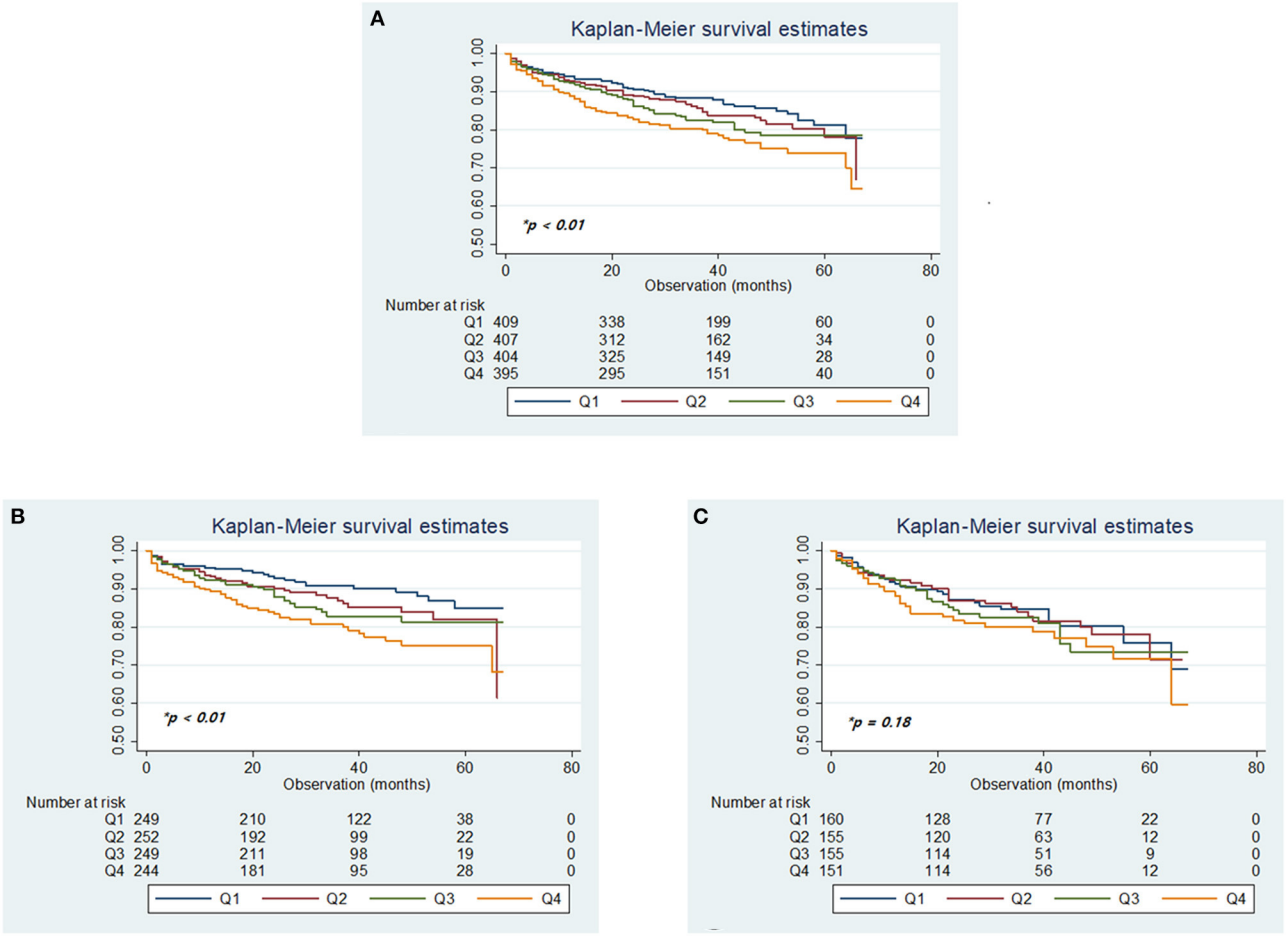
Kaplan–Meier plots of overall survival according to the quartiles of QTc intervals in the total cohort **(A)**, in men **(B)**, and women **(C)**. *p-values determined using the log-rank test.

Over a median follow-up period of 33 months (IQR, 20–48 months), 323 (19.4%) patients died. A total of 153 deaths were stroke-related (9.2%), 32 were cardiac-related (1.9%), and there were other causes (8.3%; Supplementary Table 1), with a higher mortality rate in women (149 of 650 women [22.9%]) than men (174 of 1,018 men [17.1%]; Supplementary Figure 2). Kaplan–Meier analysis of long-term survival showed that prolonged QTc interval was dose-dependently associated with a gradually increased risk of mortality over 6 years, especially in men but not in women (Figure 3).

The crude incidence of all-cause death in the entire patient cohort increased linearly according to the increased quartiles of QTc interval. Adjusted multivariable analysis using the Cox-regression model with stepwise selection of all clinically relevant variables before adjustment for neurological severity showed that the risk of death, relative to the lowest quartile of QTc interval tended to be higher in the second and third quartiles, and significantly higher in the fourth (HR: 1.49, 95% CI: 1.07–2.08) quartile (*p* = 0.0422 for trend), before adjusting for neurological factors (Table 2).

**Table 2:** Annual incidence rate and unadjusted and adjusted hazard ratios for quartiles of sex-specific QTc intervals predicting clinical outcomes during the 6-year follow-up period.

Quartiles^*^			**Unadjusted**		Adjusted^**^		**Adjusted^†^**	
	**Number of events**	**Incidence, %/year**	**HR**	**95% CI**	**HR**	**95% CI**	**HR**	**95% CI**
**Total cohort**								
Q1	63/416 (15.1)	4.7	Reference		Reference		Reference	
Q2	72/418 (17.2)	6.1	1.22	0.87–1.71	1.16	0.82–1.65	1.17	0.82–1.66
Q3	82/418 (19.6)	7.2	1.43	1.03–1.98	1.01	0.71–1.44	0.91	0.64–1.31
Q4	106/416 (25.5)	9.7	1.89	1.39–2.59	1.49	1.07–2.08	1.18	0.84–1.66
P for trend^a^			<0.01		0.04		0.60	
**Male patients**								
Q1	31/254 (12.2)	3.8	Reference		Reference		Reference	
Q2	37/255 (14.5)	5.2	1.27	0.79–2.05	1.19	0.72–1.96	1.15	0.70–1.92
Q3	45/255 (17.6)	6.2	1.54	0.98–2.44	1.16	0.70–1.90	1.06	0.64–1.76
Q4	61/254 (24.0)	8.9	2.18	1.42–3.36	1.60	0.99–2.57	1.16	0.71–1.90
P for trend^a^			<0.01		0.06		0.65	
**Female patients**								
Q1	32/162 (19.8)	6.4	Reference		Reference		Reference	
Q2	35/163 (21.5)	7.7	1.18	0.73–1.91	1.14	0.69–1.90	1.23	0.74–2.04
Q3	37/163 (22.7)	9.1	1.33	0.83–2.14	0.86	0.50–1.47	0.81	0.48–1.38
Q4	45/162 (27.8)	11.1	1.63	1.03–2.56	1.39	0.85–2.28	1.18	0.71–1.96
P for trend^a^			0.03		0.34		0.82	

* *QTc cut-off points between quartiles 1 and 2, 2 and 3, and 3 and 4 were 427, 453, and 479 msec, respectively, for men and 444, 469, and 498 msec, respectively, for women*.

*CI, confidence interval; HR, hazard ratio*.

** *Model 1, adjusted for age, sex, conventional risk factors, comorbidities, and all laboratory results*.

† *Model 2, adjusted for all variables in model 1 plus the NIHSS score*.

a *P for trend values were calculated by treating quartiles of sex-specific QTc as continuous variables*.

To assess the cut-off value for the QTc interval prolongation that was predictive of long-term mortality, the QTc intervals were dichotomized using various cut-off values from the highest median to the decile values. The prognostic value of the highest sceptile of QTc interval prolongation (defined as ≥ 501 ms in men and ≥ 517 ms in women, HR: 1.33, 95% CI: 1.00–1.62) was significantly associated with the risk of overall mortality after adjusting for all clinically relevant variables, such as neurological factors with the highest c-index (0.848; Supplementary Table 2).

### Age and Sex Difference in QTc Interval of Stroke Patients

**Figure 4:**
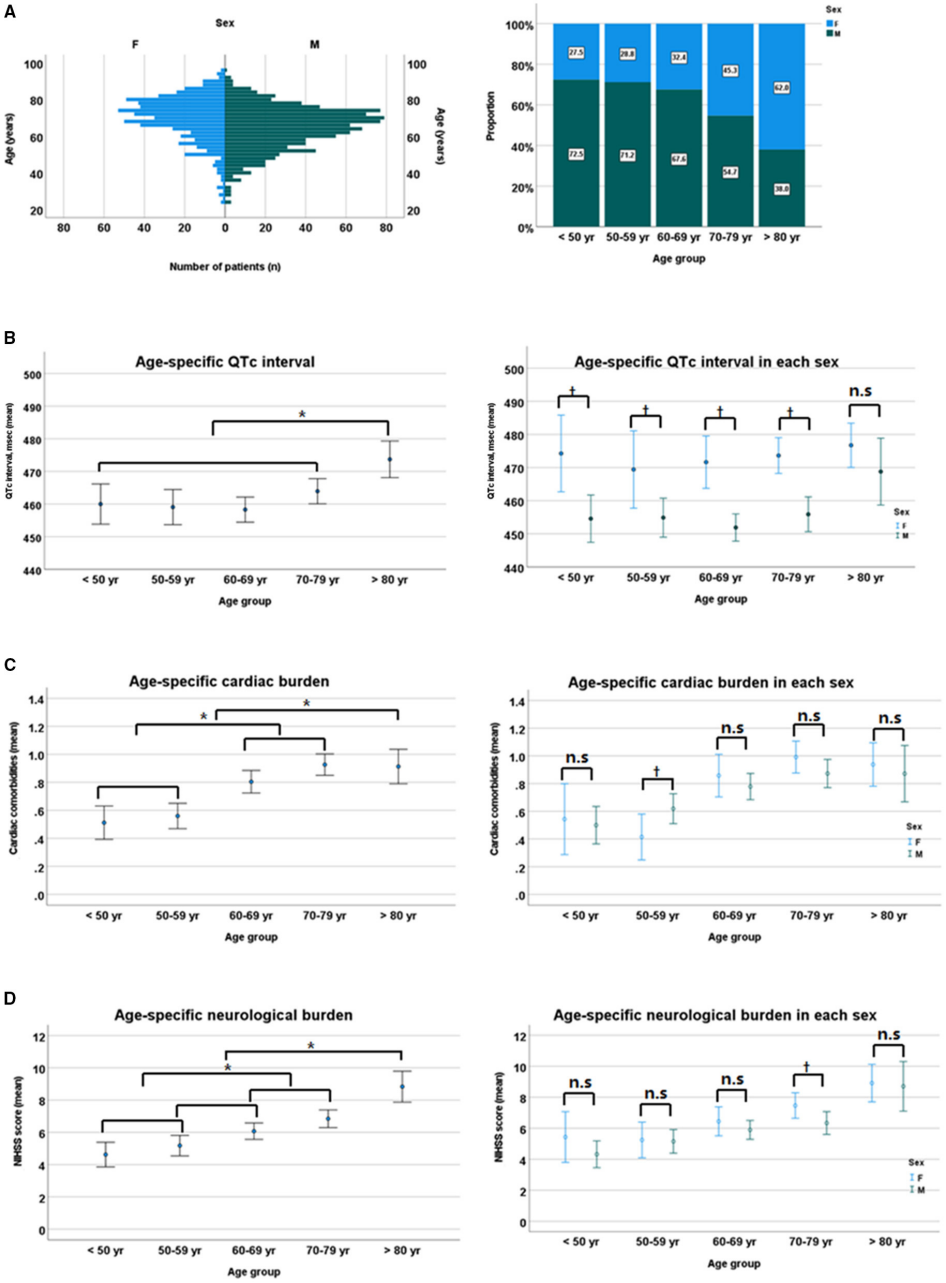
Sex-specific age distribution **(A)**, sex differences in QTc intervals **(B)**, cardiac burden **(C)**, and neurological burden **(D)**. NIHSS, National Institutes of Health Stroke Scale. *p < 0.05 by ANOVA with Duncan *post-hoc* test according to age group. ^†^*p* < 0.05 by Student *t*-test between sexes in each age group. n.s, non specific.

For the assessment of the relationship between sex and age distribution, and their impact on QTc interval, overall mean age was significantly higher in women than in men (69.3 ± 12.6 vs. 64.0 ± 11.8 years, *p* < 0.01 by Student *t*-test), with a positively skewed trend to increasing age, especially in women. In addition, women patients had a higher cardiac and neurological burden (mean number of cardiac comorbidities [0.84 ± 0.92 vs. 0.74 ± 0.86, *p* = 0.03] and mean NIHSS score [7.1 ± 6.4 vs. 5.9 ± 6.0, *p* < 0.01]) than men patients. When categorized by age group, QTc interval and the burden of cardiac and neurological conditions gradually increased with age. However, the sex-related gap of QTc interval in each age group remained consistent, although it tended to narrow gradually with age, despite the absence of significant sex difference in cardiac and neurological burdens (Figure 4).

## Discussion

This study found that the baseline QTc interval in patients with AIS was associated with the cumulative burden of cardiac comorbidities and the severity of the stroke, as well as with long-term mortality. The median QTc interval in the total cohort of 1,668 patients with a mean age of 66.0 ± 12.4 years and men predominance (61.1%) was 460.5 ms (IQR 434, 487 ms) and was normally distributed. Despite the heterogeneity in patient characteristics and the cut-off values for defining QTc interval prolongation, these results were in good agreement with a systematic review of ECG changes in patients with acute stroke (7): QTc was prolonged in at least 25% of stroke patients, such as those with hemorrhagic and ischemic stroke, and has been reported in 23–45% of patients with AIS, constituting the most frequent single ECG abnormality.

In this study, the highest quartile of the QTc interval prolongation was associated with the long-term mortality and the highest septile of QTc interval as dichotomized QTc interval prolongation was significantly associated with the long-term mortality even across stroke severity, performing better performance than other cut-off values. A meta-analysis that included multiple studies found that QTc interval > 450 ms was significantly associated with all-cause and coronary heart disease mortality in general populations despite methodological heterogeneity across studies (26). Moreover, a prolonged QT interval in patients with acute stroke was associated with a significantly greater risk of all-cause mortality within 3 months (27). The prognostic significance of prolonged QTc interval may be attributable to an increased risk of fatal or non-fatal sustained arrhythmia, as well as representing the dysregulation of the autonomic nervous system that contributes to long-term atherosclerotic changes in multiple vessels (23, 28). However, studies that included a large number of individuals undergoing medical screening found that QTc in the general population conforms to a normal Gaussian normal distribution and suggest the abnormal QTc values longer than the 97.5th percentile (i.e., >440–450 ms in men and >460 ms in women) (29). In the present study, the mean QTc interval was almost 50 ms longer than the range of QTc intervals in normal populations, a difference that may be attributed to the older age and greater burden of cardiac comorbidities and neurological stress as well as medications contributing QTc interval prolongation in stroke patients than in normal populations. Thus, an appropriate cut-off value defining QTc prolongation in stroke patients should, therefore, be higher than in a normal population.

The present study also found that the quartiles of QTc interval were related to the burdens of cardiac and neurological conditions and the crude incidence of all-cause death, regardless of sex. QTc interval prolongation represents a delay in ventricular repolarization and is associated with various etiologies, such as VH, IHD, certain drugs, dyselectrolytemia, hypertension, diabetes, and stroke (30). QTc interval prolongation also indicates a dysregulated autonomic nervous system, which leads to long-term atherosclerotic changes in systematic vessels and increases the risks of cerebro-cardiovascular diseases and mortality (31). Similarly, abrupt induction of autonomic dysregulation resulting from over-activity of the sympathetic nervous system during acute stroke may prolong QTc interval in patients with AIS (32). Alternatively, direct neuronal effects mediated by the central nervous system *via* neuron endings on the heart or coexisting cardiac abnormalities may also play a role (16, 23). Thus, a prolonged QTc interval may reflect both the direct and indirect cerebral effects on preexisting cardiac comorbidities and can be regarded as a surrogate for cardiovascular and cerebrovascular risk profiles in patients with AIS. Furthermore, our study revealed no significant difference in the proportion of medications affecting the QTc interval among the different quartiles of the QTc interval (Table 3), which can reduce the risk of heterogeneity of our results.

**Table 3:** Medications affecting QTc interval according to the quartiles of the QTc interval.

	**Quartiles of QTc interval**				
**Variable**	**Q1 (*n* = 416)**	**Q2 (*n* = 418)**	**Q3 (*n* = 418)**	**Q4 (*n* = 416)**	***P-*value^†^**
QTc interval prolongation					
ACEIs or ARBs	119 (28.6)	130 (31.1)	134 (32.1)	118 (28.4)	0.57
Calcium channel blockers	111 (26.7)	109 (26.1)	112 (26.8)	126 (30.3)	0.51
Beta blockers	76 (18.3)	63 (15.1)	73 (17.5)	72 (17.3)	0.65
Digoxin	12 (2.9)	12 (2.9)	13 (3.1)	14 (3.4)	0.97
Other QTc interval prolonging drugs^*^	57 (13.7)	54 (12.9)	65 (15.6)	62 (14.9)	0.70
Anti-QTc interval prolongation (33)					
Statin	69 (16.6)	83 (19.9)	85 (20.3)	86 (20.7)	0.42

*Variables are presented as number (%)*.

*ACEI, angiotensin-converting enzyme inhibitor; ARB, angiotensin receptor blocker*.

* *QTc interval prolonging drugs determined by the Anatomical Therapeutic Chemical (ATC) code include cilostazol (B01AC23), domperidone (A03FA03), flecainide (C01BC04), amiodarone (C01BD01), sotalol (C07AA07), nicardipine (C08CA04), solifenacin (G04BD08), azithromycin (J01FA10), ofloxacin (J01MA01), ciprofloxacin (J01MA02), tamoxifen (L02BA01), tacrolimus (L04AD02), tizanidine (M03BX02), amantadine (N04BB01), quetiapine (N05AH04), lithium (N05AN01), risperidone (N05AX08), fluoxetine (N06AB03), citalopram (N06AB04), sertraline (N06AB06), escitalopram (N06AB10), galantamine (N06DA04), imipramine (N06AA02, N06AA02), amitriptyline (N06AA09), and diphenhydramine (R06AA02), as listed at www.qtdrugs.org*.

† *p-values were calculated by Pearson chi-square test*.

The disproportionate age distribution observed in our patients was affected by sex differences, as well as affecting QTc interval and clinical characteristics in patients with stroke. The present study found that women patients were older, had a higher cardiac and neurological burden than men patients, with QTc interval being more prolonged in women than men pa gap of 20 ms in QTc interval. This finding was in agreement with the previous studies showing sex gaps of 6–10 ms in older age groups and 12–15 ms in younger adults (17), with the gap narrowing after age 40 years (29). However, we observed no sex difference in cardiac and neurological burdens across age groups. These findings confirm that QTc interval can be regarded as a surrogate for cardiovascular and cerebrovascular risk profiles, and eventually for long-term mortality, across the sex and age distribution.

### Limitations

This study had several limitations. First, the study was performed in a single center, which may reduce its generalizability. In addition, we were unable to determine non-fatal long-term outcomes, such as major adverse cerebro-cardiovascular events (MACCE), because follow-up information about patients was obtained using the national death certificate data from the Korean National Statistical Office. However, this study enrolled all consecutive patients with AIS within 24 h of stroke onset without excluding patients with concomitant cardiac comorbidities or those taking a wide range of medications contributing QTc prolongation. Thus, our results reflect a real-world clinical situation of QTc interval changes in AIS patients. Second, the mechanism underlying QTc interval prolongation remains tentative because ECG was performed at single time points, not overtime. Furthermore, major cardiac and non-cardiac comorbidities were defined as the previous history and/or ECG results at admission, thus possibly underestimating subclinical conditions. In addition, the contribution of each cardiac and non-cardiac condition to QTc interval prolongation is not equally the same, thus a composite term of “cardiac and neurological burdens” is rather an arbitrary one. To overcome these problems, we are currently conducting a prospective trial with serial measurements of troponin and ECG in patients with AIS (Clinical implications of elevated cardiac troponin-I elevation in acute stroke patients; KCT0000682; https://cris.nih.go.kr/cris), which reveal serial changes in QTc intervals and their relationship to major adverse cerebro-cardiovascular events in patients with AIS, as well as the value of troponin (34). Furthermore, this prospective study includes measurements of disease-specific biomarkers such as AF, IHD, and VH, and ECG performed three times during hospitalization to improve the detection rate of cardiac comorbidities. Finally, various medications that contribute to QTc interval should be adjusted, with consideration of the dosage and duration of medication. However, the contribution of representative medications to QTc interval did not differ significantly across the quartiles of QTc intervals in our study.

### Conclusions

Prolonged QTc interval was associated with the increased risk of long-term mortality, in parallel with the increasing trend of the prevalence of cardiovascular risk profiles and stroke severity, across sex differences, such as different distributions of age, comorbidities, morality, and QTc intervals in AIS patients.

## Data Availability Statement

The raw data supporting the conclusions of this article will be made available by the authors, without undue reservation.

## Ethics Statement

The study protocol was approved by the Institutional Review Board of the Asan Medical Center. Written informed consent for participation was not required for this study in accordance with the national legislation and the institutional requirements.

## Author Contributions

S-HA contributed to the study concept, study design, data analysis and interpretation, and drafting and revising the manuscript. J-SL and M-SY contributed to the conception of the study, data analysis, and statistics. Y-HK, M-GP, and K-PP contributed to the conception of the study, data analysis, and acquisition of clinical data. M-SY, J-HH, and S-YK contributed to the conception of the study, data analysis, and acquisition of clinical data. D-WK and JK contributed to the conception of the study, data analysis, and acquisition of clinical and imaging data. SK contributed to the study concept, study design, analysis and interpretation of the imaging and clinical data, drafting and revising the manuscript, and study supervision. All authors contributed to the article and approved the submitted version.

## Funding

This study was supported by Research Institute for Convergence of Biomedical Science and Technology, Pusan National University Yangsan Hospital (20-2018-001).

## Conflict of Interest

The authors declare that the research was conducted in the absence of any commercial or financial relationships that could be construed as a potential conflict of interest.

## Publisher's Note

All claims expressed in this article are solely those of the authors and do not necessarily represent those of their affiliated organizations, or those of the publisher, the editors and the reviewers. Any product that may be evaluated in this article, or claim that may be made by its manufacturer, is not guaranteed or endorsed by the publisher.
