# Supplementary material for: Prognostic Significance of Prolonged Corrected QT Interval in Acute Ischemic Stroke
Source: Front Neurol. 2021 Dec 20;12:759822. doi: 10.3389/fneur.2021.759822 (PMC8720760; doi:10.3389/fneur.2021.759822)
Supplement: Supplementary file 1 [file Data_Sheet_1.docx]

**Supplemental materials**

**Supplemental table 1. Causes of death according to the quartiles of the QTc interval**

|  | Quartiles of QTc interval | | | |  |
| --- | --- | --- | --- | --- | --- |
| Variable | Q1  (n = 416) | Q2  (n = 418) | Q3  (n = 418) | Q4  (n = 416) | *P-*value |
| **Cause of death** |  |  |  |  |  |
| Stroke-related | 30 (7.2) | 27 (6.5) | 36 (8.6) | 60 (14.4) | < 0.01† |
| Cardiac-related | 7 (1.7) | 7 (1.7) | 8 (1.9) | 10 (2.4) | 0.86† |
| Others | 26 (6.3) | 38 (9.1) | 38 (9.1) | 36 (8.7) | 0.38† |
| **Cause of death by interval** |  |  |  |  | 0.04* |
| Stroke-related, within 3 months | 10 (2.4) | 6 (1.4) | 13 (3.1) | 25 (6.0) |  |
| Stroke-related, beyond 3 months | 20 (4.8) | 21 (5.0) | 23 (5.5) | 35 (8.4) |  |
| Cardiac-related, within 3 months | 2 (0.5) | 3 (0.7) | 4 (1.0) | 5 (1.2) |  |
| Cardiac-related, beyond 3 months | 5 (1.2) | 4 (1.0) | 4 (1.0) | 5 (1.2) |  |
| Others, within 3 months | 7 (1.7) | 14 (3.3) | 10 (2.4) | 9 (2.2) |  |
| Others, beyond 3 months | 19 (4.6) | 24 (5.7) | 28 (6.7) | 27 (6.5) |  |

Variables are presented as number (%).

†*p*-values were calculated by Pearson chi-square test.

**p*-values were calculated by Chi square likelihood test.

**Supplemental table 2. Comparison of the annual incidence rate and unadjusted and adjusted hazard ratios for dichotomized QTc interval to predict clinical outcomes during the 6-year follow-up period**

|  |  |  |  |  | Unadjusted | |  | Adjusted^*^ | |  | Adjusted† | |
| --- | --- | --- | --- | --- | --- | --- | --- | --- | --- | --- | --- | --- |
|  |  | Number of events, | Incidence, % |  | HR | 95% CI |  | HR | 95% CI |  | HR | 95% CI |
| **^a^By the median in each sex** | | | |  |  |  |  |  |  |  |  |  |
| QTc prolongation (-) |  | 135/834 (16.2) | 5.4 |  | Reference | |  | Reference | |  | Reference | |
| QTc prolongation (+) |  | 188/834 (22.5) | 8.4 |  | 1.50 | 1.20–1.87 |  | 1.14 | 0.90–1.45 |  | 0.96 | 0.75–1.23 |
|  |  |  |  |  | (c-index: 0.557) | |  | (c-index: 0.809) | |  | (c-index: 0.846) | |
| **^b^By the highest tertile in each sex** | | | |  |  |  |  |  |  |  |  |  |
| QTc prolongation (-) |  | 185/1108 (16.7) | 5.7 |  | Reference | |  | Reference | |  | Reference | |
| QTc prolongation (+) |  | 138/560 (24.6) | 9.4 |  | 1.59 | 1.27–1.98 |  | 1.35 | 1.06–1.73 |  | 1.11 | 0.86–1.43 |
|  |  |  |  |  | (c-index: 0.561) | |  | (c-index: 0.810) | |  | (c-index: 0.847) | |
| **^c^By the highest quartile in each sex** | | | |  |  |  |  |  |  |  |  |  |
| QTc prolongation (-) |  | 217/1252 (17.3) | 6.0 |  | Reference | |  | Reference | |  | Reference | |
| QTc prolongation (+) |  | 106/416 (25.5) | 9.7 |  | 1.57 | 1.24–1.97 |  | 1.42 | 1.09–1.84 |  | 1.17 | 0.89–1.52 |
|  |  |  |  |  | (c-index: 0.552) | |  | (c-index: 0.810) | |  | (c-index: 0.847) | |
| **^d^By the highest quintile in each sex** | | | |  |  |  |  |  |  |  |  |  |
| QTc prolongation (-) |  | 238/1335 (17.8) | 6.2 |  | Reference | |  | Reference | |  | Reference | |
| QTc prolongation (+) |  | 85/333 (25.5) | 10.0 |  | 1.55 | 1.21–1.98 |  | 1.55 | 1.18–2.04 |  | 1.22 | 0.92–1.61 |
|  |  |  |  |  | (c-index: 0.547) | |  | (c-index: 0.811) | |  | (c-index: 0.847) | |
| **^e^By the highest sextile in each sex** | | | |  |  |  |  |  |  |  |  |  |
| QTc prolongation (-) |  | 246/1384 (17.8) | 6.1 |  | Reference | |  | Reference | |  | Reference | |
| QTc prolongation (+) |  | 77/284 (27.1) | 11.0 |  | 1.69 | 1.31–2.19 |  | 1.54 | 1.16–2.04 |  | 1.21 | 0.91–1.62 |
|  |  |  |  |  | (c-index: 0.551) | |  | (c-index: 0.812) | |  | (c-index: 0.847 | |
| **^f^By the highest septile in each sex** | | | |  |  |  |  |  |  |  |  |  |
| QTc prolongation (-) |  | 253/1430 (17.7) | 6.1 |  | Reference | |  | Reference | |  | Reference | |
| QTc prolongation (+) |  | 70/238 (29.4) | 12.4 |  | **1.89** | **1.45–2.46** |  | **1.74** | **1.30–2.32** |  | **1.33** | **1.00–1.80** |
|  |  |  |  |  | (c-index: 0.555) | |  | (c-index: 0.813) | |  | (c-index: 0.848) | |
| **^g^By the highest octile in each sex** | | | |  |  |  |  |  |  |  |  |  |
| QTc prolongation (-) |  | 260/1460 (17.8) | 6.1 |  | Reference | |  | Reference | |  | Reference | |
| QTc prolongation (+) |  | 63/208 (30.3) | 13.0 |  | 1.96 | 1.49–2.58 |  | 1.65 | 1.22–2.24 |  | 1.27 | 0.92–1.73 |
|  |  |  |  |  | (c-index: 0.552) | |  | (c-index: 0.812) | |  | (c-index: 0.848) | |
| **^h^By the highest decile in each sex** | | | |  |  |  |  |  |  |  |  |  |
| QTc prolongation (-) |  | 264/1483 (17.8) | 6.1 |  | Reference | |  | Reference | |  | Reference | |
| QTc prolongation (+) |  | 59/185 (31.9) | 14.0 |  | 2.09 | 1.57–2.77 |  | 1.77 | 1.30–2.42 |  | 1.32 | 0.96–1.82 |
|  |  |  |  |  | (c-index: 0.552) | |  | (c-index: 0.813) | |  | (c-index: 0.848) | |
| **^i^QTc prolongation defined by community-based populations** | | | |  |  |  |  |  |  |  |  |  |
| QTc prolongation (-) |  | 109/723 (15.1) | 4.9 |  | Reference | |  | Reference | |  | Reference | |
| QTc prolongation (+) |  | 214/945 (22.6) | 8.6 |  | 1.65 | 1.31–2.08 |  | 1.25 | 0.98–1.60 |  | 1.06 | 0.83–1.37 |
|  |  |  |  |  | (c-index: 0.567) | |  | (c-index: 0.810) | |  | (c-index: 0.847) | |

CI = confidence interval; HR = hazard ratio.

*Model 1, adjusted for age, sex, conventional risk factors, comorbidities, and all laboratory results in table 1.

†Model 2, adjusted for all variables in model 1 plus NIHSS score.

^a^QTc prolongation was defined as a QTc interval ≥ 454 ms in men and ≥ 470 ms in women, based on the median for each sex.

^b^QTc prolongation was defined as a QTc interval ≥ 469 ms in men and ≥ 487 ms in women, based on the highest tertile in each sex.

^c^QTc prolongation was defined as a QTc interval ≥ 479 ms in men and ≥ 498 ms in women, based on the highest quartile in each sex.

^d^QTc prolongation was defined as a QTc interval ≥ 488 ms in men and ≥ 505 ms in women based on the highest quintile in each sex.

^e^QTc prolongation was defined as a QTc interval ≥ 495 ms in men and ≥ 510 ms in women based on the highest sextile in each sex.

^f^QTc prolongation was defined as a QTc interval ≥ 501 ms in men and ≥ 517 ms in women based on the highest septile in each sex.

^g^QTc prolongation was defined as a QTc interval ≥ 507 ms in men and ≥ 519 ms in women based on the highest octile in each sex.

^h^QTc prolongation was defined as a QTc interval ≥ 510 ms in men and ≥ 523 ms in women based on the highest decile in each sex.

^i^QTc prolongation was defined as a QTc interval ≥ 450 ms in men and ≥ 460 ms in women based on the generally adopted cut-off values in a community-based population.

**Figure Legends**

**Supplemental figure 1. Distribution of overall and sex-specific QTc intervals**


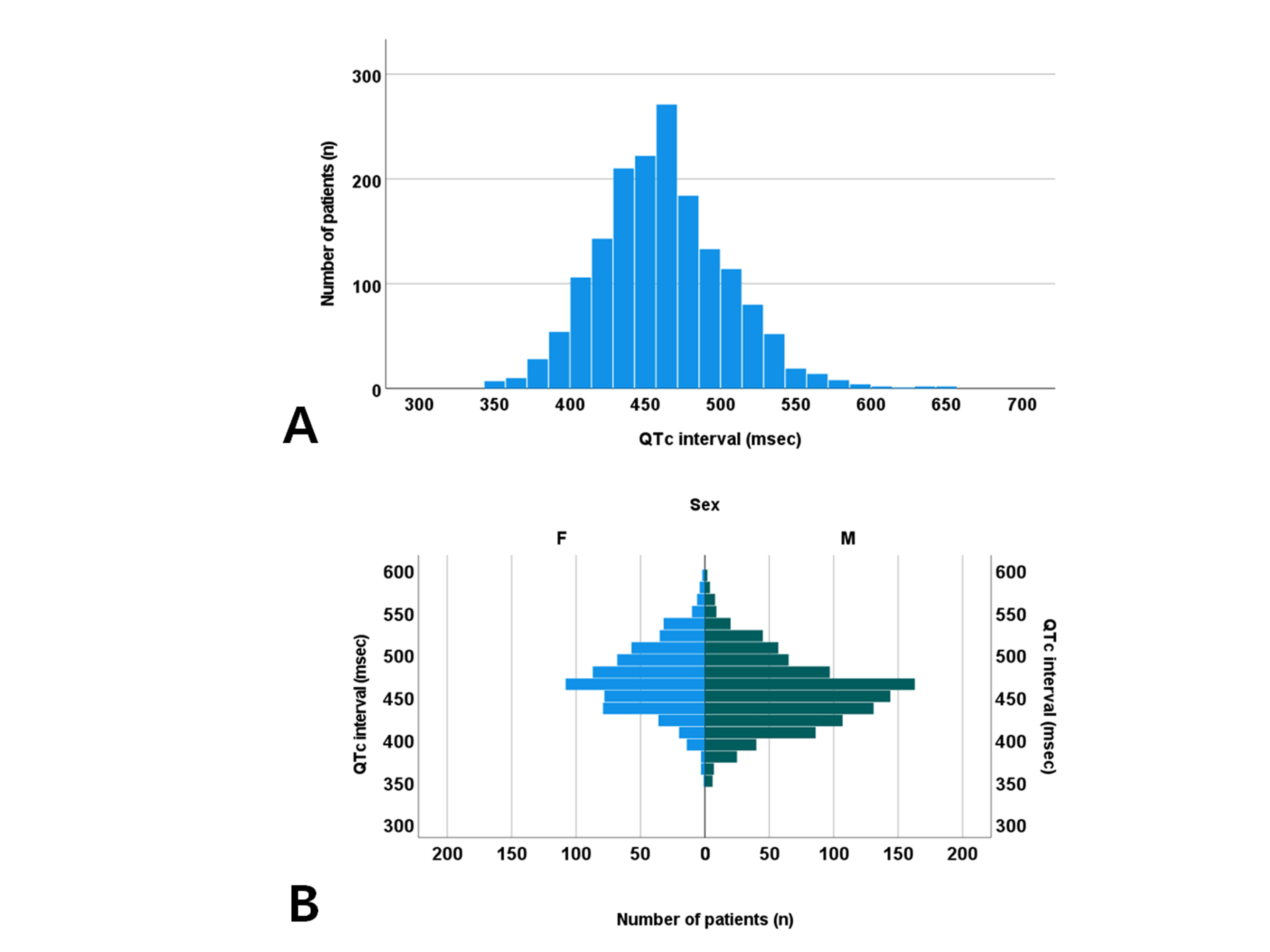


**Supplemental figure 2. Kaplan–Meier plots of overall survival for the different sexes**

**
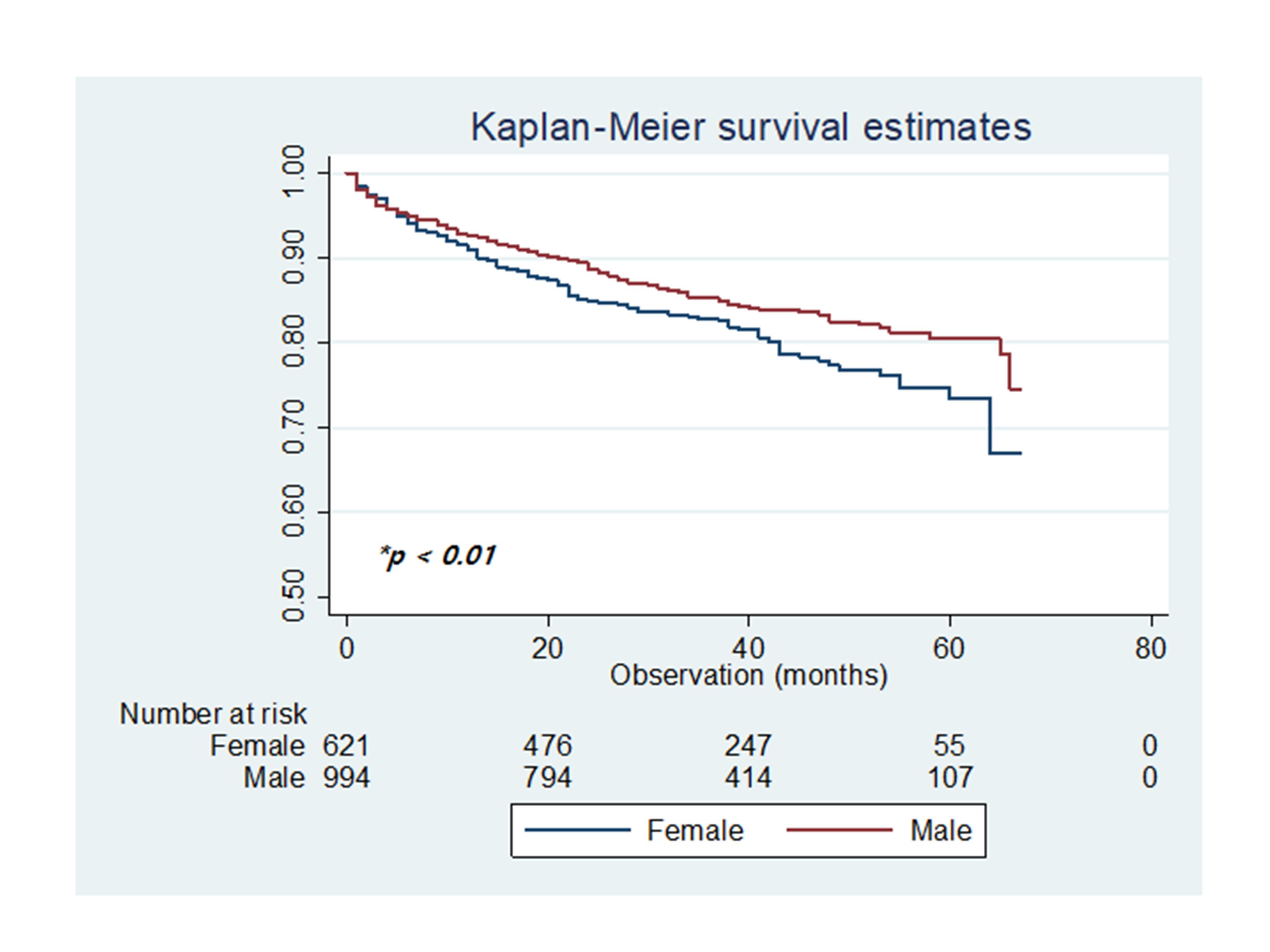
**
